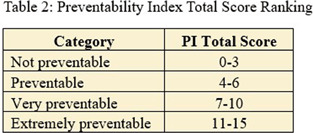# Development of a CLABSI Preventability Index to Target Improvement Efforts

**DOI:** 10.1017/ash.2025.285

**Published:** 2025-09-24

**Authors:** Karen Brust, Angie Dains, Holly Meacham, Mary Beth Hovda-Davis, Elizabeth Krigbaum, Kristin Varzavand, Olivia Wulf

**Affiliations:** 1 University of Iowa Health Care; 2 University of Iowa Hospital and Clinics; 3 University of Iowa Hospital and Clinics; 4 University of Iowa Healthcare; 5 University of Iowa Healthcare; 6 University of Iowa Health Care; 7 University of Iowa Health Care

## Abstract

**Background:** Central Line-Associated Bloodstream Infections (CLABSI) are multifactorial, making trends difficult to identify. CLABSI can occur from the time of insertion to delayed removals beyond the time central access was indicated. The objective of creating a CLABSI Preventability Index tool was to enable strategic quality improvement work. **Methods:** A preventability index tool was created with stakeholder input and was categorized into four categories (see Table 1): Indication for Line, Care and Maintenance and Line Access, Diagnostic Stewardship, and Specimen Collection. Each category had one or more questions prompting users to assign points for each preventable action. Scores range from 0 through 15, with the higher score indicating more prevention opportunities. (See table 2). **Results:** During the 2024 calendar year, there were 25 Adult CMS CLABSIs. The preventability index was applied to each case. There was 1 ‘extremely preventable’ case, 2 ‘very preventable’ cases, 6 ‘preventable’ cases and 16 ‘not preventable’ cases. In the 3 cases scoring very preventable or extremely preventable, the category “indication for line” was consistently scored high. Two of the 3 cases had preventable actions from a care and maintenance standpoint, 2 cases scored for diagnostic stewardship category and all 3 cases scored in the specimen collection category. In the 22 cases scoring 6 or lower, 0 scored in the indication for line category, 16 scored in the care and maintenance category, 11 scored in diagnostic stewardship and 4 scored in specimen collection. **Conclusion:** The preventability index objectively identifies the highly preventable CLABSIs in order to target high-priority actions to prevent future cases. Based on this tool, the use of central lines when not indicated causes the highest preventability scores, but care and maintenance activities score the most frequently.

**Figure:**
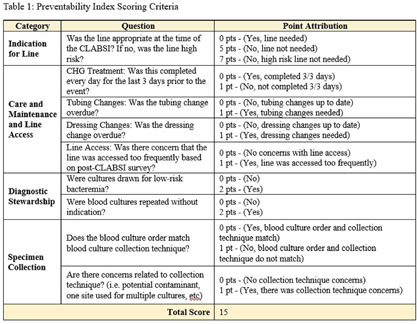

**Figure:**